# Technical Section

**DOI:** 10.1308/003588412X13373405385214a

**Published:** 2012-07

**Authors:** Bruce Campbell

## BACKGROUND

Wrong level spine surgery dominates malpractice claims.^1^ In one series, the wrong level was approached in 15% of cases undergoing a lumbar discectomy.^2^ We report three steps to avoid incorrect level surgery and litigation.

## TECHNIQUE

**Figure 1:**
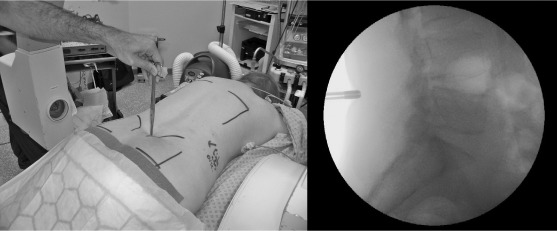
Radiopaque marker used to confirm disc level

**Figure 2:**
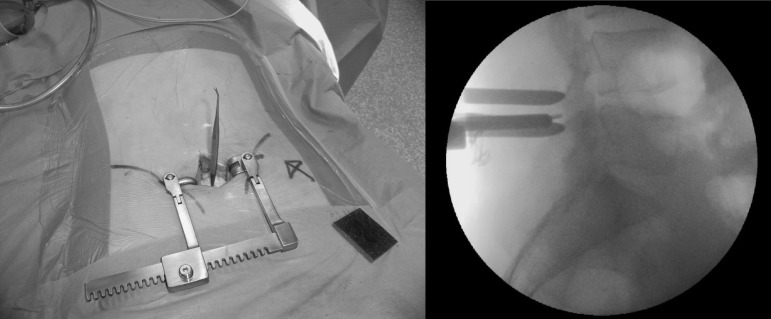
Confirmation of level prior to discectomy

**Figure 3:**
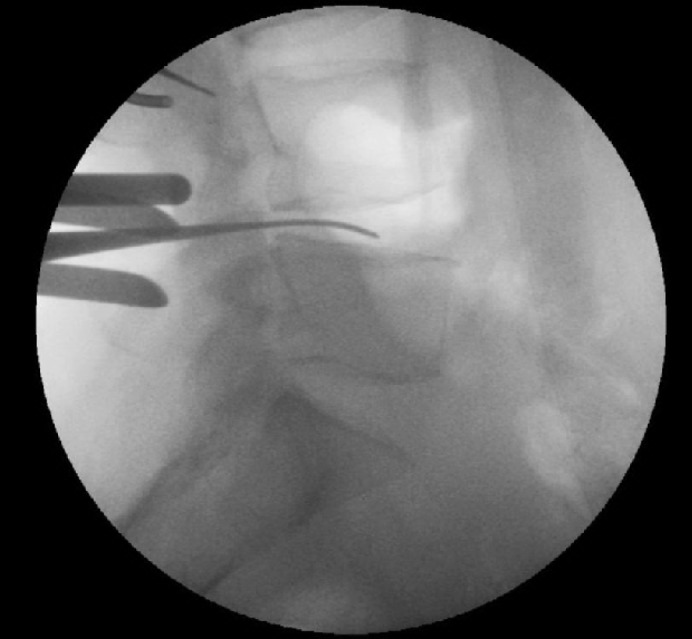
Correct level discectomy performed

Prior to skin incision, lateral fluoroscopy is performed with the patient prone. A radiopaque pointer is used to identify the level of the disc being approached (Fig 1) and this level is marked on the skin. The surgical approach is centred on the skin mark and once the laminae have been exposed, a radiopaque instrument is inserted below the inferior border of the upper lamina, at the level of the disc. Further fluoroscopy is used to confirm correct positioning (Fig 2). A final image is taken following discectomy with an instrument in the disc space, thereby confirming correct level surgery (Fig 3).

## DISCUSSION

In 2010 a retrospective study by Irace and Corona reported the use of a pre-incision wire marker inserted to the spinous process using radiography for patients undergoing microlumbar discectomies.^3^ They described one case of incorrect approach. We have defined a reliable technique that confirms with absolute certainty the correct level for discectomy before, during and after the procedure. We have performed 64 open lumbar discectomies using this technique with no cases of incorrect level discectomy.

## References

[1] Fager CA. Malpractice issues in neurological surgery. Surg Neurol 2006; 65: 416–421 16531218 10.1016/j.surneu.2005.09.026

[2] Ammerman JM, Ammerman MD, Dambrosia J, Ammerman BJ. A prospective evaluation of the role for intraoperative x-ray in lumbar discectomy. Predictors of incorrect level exposure. Surg Neurol 2006; 66: 470–473 17084188 10.1016/j.surneu.2006.05.069

[3] Irace C, Corona C. How to avoid wrong-level and wrong-side errors in lumbar microdiscectomy. J Neurosurg Spine 2010; 12: 660–665 20515352 10.3171/2009.12.SPINE09627

